# The impact of dance on the mental health of older adults: a network meta-analysis of anxiety, depression, and well-being

**DOI:** 10.3389/fpsyg.2025.1733911

**Published:** 2025-11-28

**Authors:** Yuexin Zhang, Hongtao Ma

**Affiliations:** College of Education, Beijing Sport University, Beijing, China

**Keywords:** older adults, dance, anxiety, depression, well-being, mental health

## Abstract

**Objective:**

This systematic review and meta-analysis (PROSPERO CRD420251015051) compared the effects of nine distinct dance interventions on the mental health of older adults.

**Methods:**

Six databases were searched from the earliest records to April 2025. Studies investigating a dance intervention lasting≥4 weeks, including Mental Health and cognitive function health outcomes. Two independent reviewers performed literature screening and data extraction. Review Manager 5.4 was used for pairwise meta-analyses and risk of bias assessment, while Stata 18.0 software was employed for network meta-analyses.

**Results:**

Of 269 records identified, 13 studies met the inclusion criteria. Total sample size of included studies was 1,083 (females, males). The results of the traditional meta-analysis showed that Chinese square dance was superior to the control group in alleviating depression (SMD = 1.11, 95% CI: 0.48, 1.75). For well-being, Chinese square dance was superior to the control group in enhancing well-being (SMD = −1.98, 95% CI: −2.55, −1.41). For anxiety, Chinese square dance was superior to the control group in alleviating anxiety (SMD = 1.21, 95% CI: 0.65, 1.76). For cognitive function, no significant differences were found. In the network meta-analysis, the ranking of treatment effects for depression showed that dance games > Chinese square dance > rhythmic gymnastics > Turkish folk dance > Poco dance > ballroom dance > aerobic dance > control group> creative dance. The ranking of treatment effects for well-being showed that Chinese square dance > aerobic dance > Turkish folk dance > control group. The ranking of treatment effects for anxiety showed that Chinese square dance = group dance > Poco dance > control group, while for cognitive function, the ranking showed Poco dance > Chinese square dance > creative dance > control group.

**Conclusion:**

This study found that dance has positive effects on improving depression and anxiety whilst enhancing well-being among older adults. Among the nine different types of dance interventions, it was considered an effective approach for improving depression, well-being, and anxiety. However, we encourage older adults to choose dance modalities that suit their interests to enhance adherence.

## 1 Introduction

Population aging is a key demographic trend in the 21st century, resulting from declining fertility rates, increased life expectancy, and healthcare improvements (Vaupel et al., 2021). According to the World Health Organization (WHO), the global population aged 60 years and older was 1.1 billion in 2023 (World Health Organization, 2023), representing 13.5% of the world’s total and is projected to reach 1.4 billion by 2030 and 2.1 billion by 2050 (Becker and Fanzo, 2023). This trend varies regionally: developed countries like Japan and Italy have over 29 and 25% of their populations aged 65 and older, respectively (Newmyer et al., 2022). In low- and middle-income countries, aging occurs rapidly, with 80% of the global older population expected to reside there by 2050 (Newmyer et al., 2022). In China, the population aged 60 and older reached 297 million in 2023 (21.1% of total) and is projected to exceed 366 million by 2050, straining healthcare systems (Scott, 2021; Dong et al., 2022; Lobanov-Rostovsky et al., 2023).

Aging is associated with increased risks of mental health issues (Carpenter et al., 2022), including depression, anxiety, reduced well-being, and cognitive function decline (Reynolds et al., 2022). Globally, around 14.1% of adults aged 70 and older have a mental disorder, with depression affecting 19.2% and anxiety 16.5% of older adults (Li et al., 2025b). These conditions are linked to higher mortality (Paykel, 2006; Chand and Tung, 2014), functional decline (White et al., 2016), and healthcare utilization (White et al., 2015; White et al., 2017). Anxiety disorders co-occur with depression and contribute to cognitive function impairment and reduced quality of life (Bloom et al., 2009; Wolitzky-Taylor et al., 2010; Calderón-Larrañaga et al., 2019). Well-being is affected by factors such as social isolation and loneliness, impacting 27.6% of older adults globally (Wilson et al., 2019; Lal et al., 2022). Cognitive function decline affects 5–8% of those over 60, with dementia cases estimated at 57 million worldwide in 2021, projected to reach 139 million by 2050 (Association, 2019; Gavrilova and Alvarez, 2021; Kalaria et al., 2024). Risk factors include physical inactivity and chronic stress, which interact with mental health conditions (Peçanha et al., 2020; Delerue Matos et al., 2021). In China, depression prevalence is 20%–30% and anxiety 15% among older adults (Qin et al., 2018). These issues contribute to economic costs, such as dementia exceeding US$1.3 trillion annually, and represent 15% of disability-adjusted life years in older adults (Nandi et al., 2022; Wimo et al., 2023). Limited access to mental health services—only 20% in low-income countries—worsens outcomes (Menassa et al., 2023; Kirkbride et al., 2024).

Dance interventions are non-pharmacological options for improving psychological and cognitive function health in older adults (Wu et al., 2021). Unlike other forms of exercise, dance uniquely integrates physical activity with social interaction, rhythmic synchronization, and creative expression, which may enhance motivation, social cognition, and emotional well-being more effectively (Himberg et al., 2018; Prudente et al., 2024). Systematic reviews indicate that dance reduces depression and anxiety, improves well-being, and enhances cognition through physical, social, and rhythmic elements (Koch et al., 2019; Teixeira-Machado et al., 2019; Fong Yan et al., 2024).

However, prior research has limitations. Previous reviews have not compared different dance styles using network meta-analysis, often varied in methodological quality, and did not consistently include well-being as an outcome. Intervention formats are heterogeneous (e.g., durations from 4 to 24 weeks), limiting comparative efficacy assessments. Evidence for clinical populations remains limited, it is unclear which dance type is most effective for specific outcomes. This Network Meta-Analysis (NMA) synthesizes randomized controlled trials to compare dance formats, addressing these gaps and providing evidence-based recommendations for geriatric mental health.

## 2 Methods

### 2.1 Eligibility criteria

This study was prospectively registered on the PROSPERO platform (registration number: CRD420251015051). The eligibility criteria were defined in accordance with the PICOS framework.

- Population: Older adults aged 60 years or older, without restrictions on gender, ethnicity, or comorbidities, excluding those with severe cognitive function impairment (e.g., dementia) that precluded participation in dance interventions.
- Intervention: Any form of dance-based interventions, including but not limited to structured dance programs (e.g., square dance, ballroom dance), lasting at least 4 weeks, with a minimum frequency of once per week.
- Comparison: Control groups involving no intervention, usual care, or alternative non-dance physical activities.
- Outcomes: Primary outcomes included depression, well-being and anxiety. Secondary outcomes encompassed cognitive function.
- Study Design: RCTs or quasi-experimental studies published in peer-reviewed journals, with no language restrictions, from database inception to April 2025.

### 2.2 Information retrieval

Two independent reviewers conducted literature searches across PubMed, Web of Science, EBSCO, ScienceDirect, the China National Knowledge Infrastructure (CNKI) database, and the Wanfang Data Knowledge Service Platform. A second reviewer independently verified the search results to ensure completeness and minimize bias, in accordance with PRISMA guidelines. Search terms included: “Dance”, “Dance Therapy”, “Tango”, “Latin Dance”, “Folk Dance”, “Street dance”, “Line dance”, “Intervention”, “Randomized controlled study”, “Older adults”, “elderly”, “aged”, “Depression”, “Depressive Symptoms”, “Anxiety”, “Angst”, “Nervousness”, “Psychological Well-Being”, and “Psychological Wellness”. The search period spanned from the inception of each database to April 3, 2025, with the last search conducted on April 3, 2025.

Specific search strategies for each database are as follows:

- PubMed: ((“Dance”[Mesh] OR “Dance Therapy”[Mesh] OR dance[tiab] OR “dance therapy”[tiab] OR tango[tiab] OR “latin dance”[tiab] OR “folk dance”[tiab] OR “street dance”[tiab] OR “line dance”[tiab]) AND (“Aged”[Mesh] OR “older adults”[tiab] OR elderly[tiab] OR aged[tiab]) AND (“Depression”[Mesh] OR depression[tiab] OR “depressive symptoms”[tiab] OR “Anxiety”[Mesh] OR anxiety[tiab] OR angst[tiab] OR nervousness[tiab] OR “Psychological Well-Being”[Mesh] OR “psychological well-being”[tiab] OR “psychological wellness”[tiab]) AND (“Randomized Controlled Trial”[pt] OR randomized[tiab] OR RCT[tiab] OR intervention[tiab])); Filters applied: Randomized Controlled Trial, English, Humans, Middle Aged: 45–64 years, Aged: 65 + years, 80 and over: 80 + years, from 1000/1/1–2025/4/3.
- Web of Science: ((dance OR “dance therapy” OR tango OR “latin dance” OR “folk dance” OR “street dance” OR “line dance”) AND (“older adults” OR “Older adults” OR elderly OR aged OR seniors OR geriatric OR “older people” OR “senior citizens”) AND (depression OR “depressive symptoms” OR anxiety OR angst OR nervousness OR “psychological well-being” OR “psychological wellness”) AND (randomized OR RCT OR intervention OR “randomized controlled trial”)) (Topic) AND 1000-01-01/2025-04-03 (Publication Date) and English (Languages); Document Types: Article.
- EBSCO: (DE “DANCE Therapy” OR TI (dance OR “dance therapy” OR tango OR “latin dance” OR “folk dance” OR “street dance” OR “line dance”) OR AB (dance OR “dance therapy” OR tango OR “latin dance” OR “folk dance” OR “street dance” OR “line dance”)) AND (DE “OLDER People” OR TI (“older adults” OR elderly OR aged) OR AB (“older adults” OR elderly OR aged)) AND (DE “DEPRESSION (Psychology)” OR DE “ANXIETY” OR TI (depression OR “depressive symptoms” OR anxiety OR angst OR nervousness OR “psychological well-being” OR “psychological wellness”) OR AB (depression OR “depressive symptoms” OR anxiety OR angst OR nervousness OR “psychological well-being” OR “psychological wellness”)) AND (DE “RANDOMIZED Controlled Trials” OR TI (randomized OR RCT OR intervention OR “randomized controlled trial”) OR AB (randomized OR RCT OR intervention OR “randomized controlled trial”)); Filters applied: Language: English, Publication date: 1000-01-01/2025-04-03.
- ScienceDirect: TITLE-ABSTR-KEY ((“dance” OR “dance therapy” OR tango OR “latin dance” OR “folk dance” OR “street dance” OR “line dance” OR dance) AND (“older adults” OR “Older adults” OR elderly) AND (“depression” OR “depressive symptoms” OR anxiety OR angst OR nervousness OR “psychological well-being” OR “psychological wellness”)); Filters applied: Article type: Research articles; Publication Date: up to 2025-04-03.
- CNKI: (SU = ‘舞蹈’ OR SU = ‘舞蹈疗法’ OR SU = ‘探戈’ OR SU = ‘拉丁舞’ OR SU = ‘民族舞’ OR SU = ‘街舞’ OR SU = ‘排舞’) AND (SU = ‘老年人’ OR SU = ‘老年’ OR SU = ‘老年人群’) AND (SU = ‘抑郁’ OR SU = ‘抑郁症状’ OR SU = ‘焦虑’ OR SU = ‘焦虑症’ OR SU = ‘神经质’ OR SU = ‘心理福祉’ OR SU = ‘心理健康’) AND (SU = ‘干预’ OR SU = ‘随机对照试验’ OR SU = ‘RCT’).时间范围至2025年4月3日.
- Wanfang: (关键词 = (舞蹈 + 舞蹈疗法 + 探戈 + 拉丁舞 + 民族舞 + 街舞 + 排舞)) AND (关键词 = (老年人 + 老年 + 老年人群)) AND (关键词 = (抑郁 + 抑郁症状 + 焦虑 + 焦虑症 + 神经质 + 心理福祉 + 心理健康)) AND (关键词 = (干预 + 随机对照试验 + RCT)). 时间范围至2025年4月3日.

### 2.3 Inclusion and exclusion criteria

#### 2.3.1 Inclusion criteria

(1) Study type: Only randomized controlled trials (RCTs) were included; quasi-experimental designs or other non-randomized studies were excluded to minimize selection bias and ensure high methodological quality; (2) Study population: Individuals aged ≥60 years, including those with mild cognitive impairment (MCI). Cognitive function status in the included studies was assessed using standardized tools, such as the Mini-Mental State Examination (MMSE) or Montreal Cognitive Assessment (MoCA). During data extraction, eligibility was confirmed by verifying that participants exhibited no significant cognitive function deficits, defined as an average MMSE score greater than 24 or explicit exclusion of moderate-to-severe dementia in the original studies; (3) Intervention measures: The experimental group received dance intervention or dance therapy, encompassing tango, folk dance, square dancing, Latin dance, street dance, line dancing, and other dance forms. The control group underwent routine training/no intervention/walking. (4) Outcome Measures: Primary outcome measures comprised depression levels, well-being levels, and anxiety assessments. Secondary outcome measures included cognitive function evaluations. All included studies were formally published peer-reviewed articles, with no preprints or “In review” status manuscripts.

#### 2.3.2 Exclusion criteria

Studies were excluded if they met any of the following: duplicate reports, conference papers, review articles, duplicate publications, quasi-experimental or non-randomized designs, or involved participants with severe cognitive function impairment (e.g., dementia, as defined by MMSE scores < 24 or clinical diagnosis of moderate-to-severe dementia) that precluded participation in dance interventions.

### 2.4 Literature screening and data extraction

Two independent reviewers systematically extracted article information according to a predefined format. The research characteristics extracted included: (1) The purpose of the meta-analysis; (2) The search strategy; (3) Inclusion and exclusion criteria; (4) A qualitative summary of the characteristics of the dance interventions studied.

### 2.5 Evaluation of literature quality

The risk of bias was assessed across five dimensions—random sequence generation, allocation concealment, blinding, completeness of data collection, and selective reporting—in accordance with the Cochrane Reviewer’s Handbook. Based on the evidence, study quality was categorized as: low risk, high risk, or unclear risk.

### 2.6 Statistical methods

We conducted a frequentist NMA using the “network” package in Stata/MP 18.0. The analysis utilized post-intervention scores (means, standard deviations [SDs], and sample sizes) for continuous outcomes to compute standardized mean differences (SMDs) as Hedges’g, which is a bias-corrected estimator of Cohen’s d. The pooled standard deviation for each pairwise comparison was calculated as √[((n1-1) × SD1^2^ + (n2-1) × SD2^2^) / (n1 + n2–2)], where n1 and n2 are sample sizes, and SD1 and SD2 are the standard deviations of the two groups. Studies that did not report standard deviations were excluded from the analysis to avoid imputation-related biases; no missing values were imputed.

Literature quality assessment utilized RoB 2. Data setup included means, SDs, and sample sizes for continuous outcomes (network setup mean sd n, study(id) trt(t) format(augment) smd). Multi-arm trials were managed within the network setup by treating multiple intervention arms as distinct treatments connected to the shared control group, avoiding double-counting through the network model’s structure. Network mapping (network map) was used for geometry visualization. Matrix size was set to 11,000 (set matsize 11,000). Random-effects models were used: inconsistency checked globally (network meta i) and via consistency (network meta c). Local inconsistency was assessed by node-splitting (network sidesplit all, tau; *p* < 0.05 indicating inconsistency). Effects were summarized in forest plots (network forest) with SMD and 95% CI. League tables (netleague) and interval plots (intervalplot, null (0)) were generated.

Data were converted to pairs (network convert pairs) for publication bias assessment via comparison-adjusted funnel plots (netfunnel _y _stderr _t1 _t2; netfunnel _y _stderr _t1 _t2, random bycomp add (lfit _stderr _ES_CEN) noalpha). Asymmetry in the funnel plots was formally tested using Egger’s regression test (metabias, egger), which assesses small-study effects potentially indicative of publication bias. No significant asymmetry was detected (*p* > 0.05 for all comparisons), suggesting low risk of publication bias. If asymmetry had been present (e.g., p < 0.05), it could indicate selective reporting of positive results or small-study effects, potentially leading to overestimation of treatment effects and requiring cautious interpretation of the NMA results, such as through sensitivity analyses or trim-and-fill adjustments.

Rankings used SUCRA with 5,000 replications (network rank min/max, all zero reps (5000) gen(prob); sucra prob.*). For negative outcomes (e.g., depression, anxiety), minimization applied (rank min); for positive (e.g., well-being, cognition), maximization (rank max). Higher SUCRA (near 100%) indicates better efficacy.

## 3 Results

### 3.1 Literature search results

**Figure 1:**
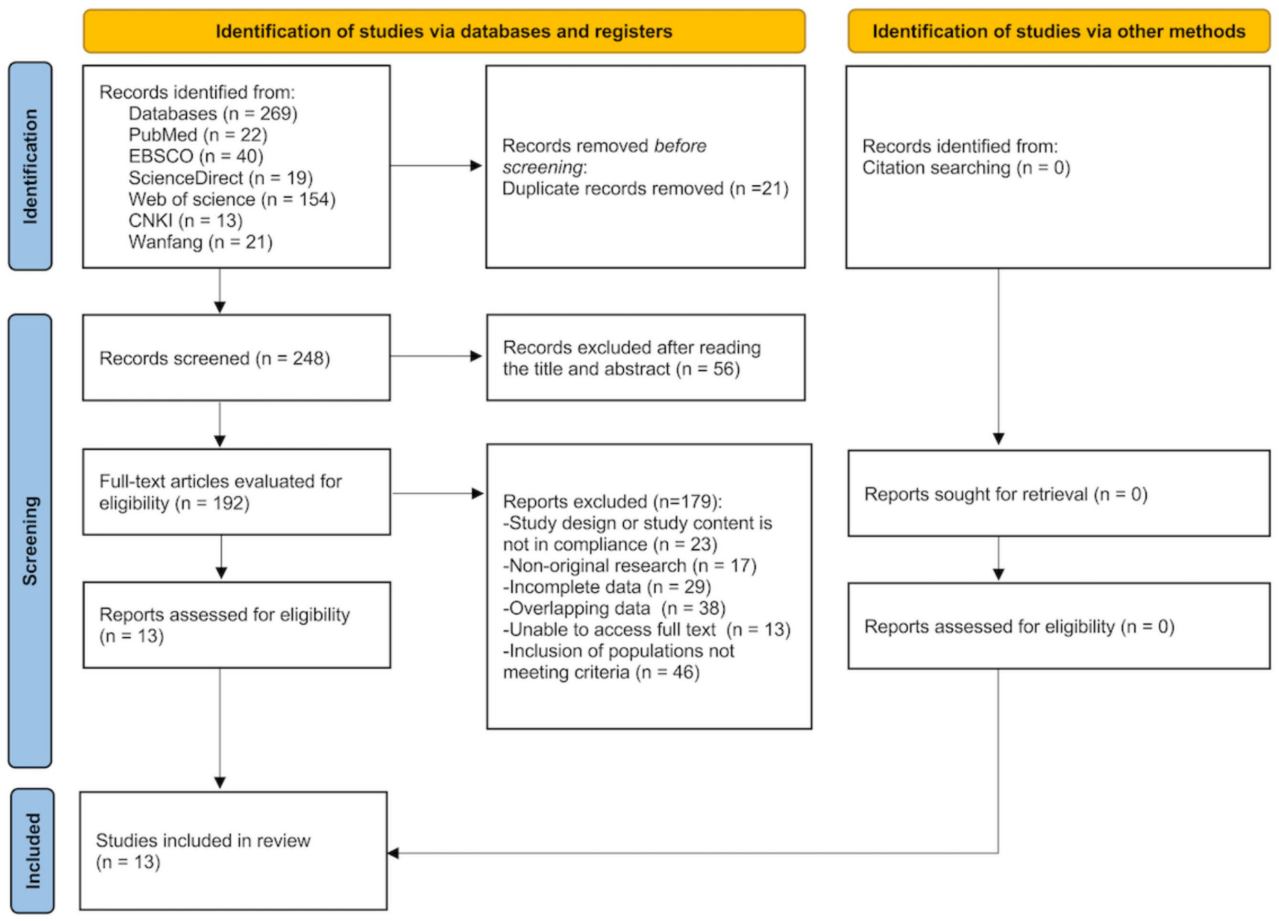
Flow diagram for study selection. Inclusion of populations not meeting criteria: Studies were excluded if they met any of the following: Duplicate reports, conference papers, review articles, duplicate publications, quasi-experimental or non-randomized designs, or involved participants with severe cognitive impairment (e.g., dementia, as defined by MMSE scores < 24, MoCA-*p* < 26 or clinical diagnosis of moderate-to-severe dementia) that precluded participation in dance interventions.

Following a database search, a total of 269 records were initially identified from various databases: PubMed (*n* = 22), EBSCO (*n* = 40), ScienceDirect (*n* = 19), Web of Science (*n* = 154), CNKI (*n* = 13), and Wanfang (*n* = 21). Prior to screening, 21 duplicate records were removed, resulting in 248 records for initial screening. After reviewing the titles and abstracts, 56 records were excluded. Subsequently, 192 full-text articles were evaluated for eligibility, leading to the assessment of 13 reports. Of these, 179 reports were excluded for the following reasons: study design or content not compliant (*n* = 23), non-original research (*n* = 17), incomplete data (*n* = 29), overlapping data (*n* = 38), unable to access full text (*n* = 13), and inclusion of populations not meeting criteria (*n* = 46). No additional records were identified through other methods, such as citation searching. Ultimately, 13 studies were included in the review. The literature screening process and outcomes are illustrated in Figure 1.

### 3.2 Basic characteristics for inclusion in the literature

The total sample size across 13 studies comprised 1,083 participants, including 861 female elderly individuals and 222 male elderly individuals. The age range was 71.97 ± 5.00 years. Regarding outcome measures, 11 studies reported on depression (Jeon et al., 2005; Eyigor et al., 2009; Zhang et al., 2012; Vankova et al., 2014; Adam et al., 2016; Wang et al., 2020; Zhao and Li, 2020; Ayari et al., 2023; Dos Santos et al., 2023; Zhang et al., 2023; Hola et al., 2024), three on anxiety (Zhang et al., 2012; Adam et al., 2016), three on well-being (Eyigor et al., 2009; Li et al., 2012; Zhang et al., 2023), and seven on cognitive function (Zhang et al., 2012; Adam et al., 2016; Wang et al., 2020; Zhao and Li, 2020; Ayari et al., 2023; Zhang et al., 2023; Hola et al., 2024). Classification based on the types of dance interventions provided in the literature yielded nine distinct dance groups alongside a control group. These comprised: Chinese square dance (Zhang et al., 2012; Wang et al., 2020; Zhao and Li, 2020; Zhang et al., 2023), Aerobic dance (Li et al., 2012; Ayari et al., 2023), Group dance (Yun-Jung et al., 2019), Rhythmic gymnastics (Jeon et al., 2005), Turkish folk dance (Eyigor et al., 2009), Ballroom dance (Vankova et al., 2014), Creative dance (Hola et al., 2024), Poco dance (Adam et al., 2016), and dance games (Dos Santos et al., 2023), as detailed in Table 1.

**Table 1:** Characteristics of the studies included in this systematic review.

Author (Year)	Country	Type	Participant characteristics				Intervention			Control	Outcome indicator
			N	Year	Health status	Female	Dance type	Dance schedule	Months		
Zhang et al. (2012)	China	RCT	60	63.3 ± 4.45	Health	53	CSD	30–60 min 4–5times/week	12	None	SECF, HAMA, HAMD
Zhao and Li (2020)	China	RCT	63	72.30 ± 5.92	MCI	52	CSD	60 min 3 times/week	3	Health education	GDS-30, MoCA-P
Li et al. (2012)	China	RCT	97	68.55 ± 4.35	Health	94	AD	50–60 min 2 times/week	3	Maintain daily activities	SF-36
Yun-Jung et al. (2019)	Korea	RCT	50	69.00 ± 0.32	Disability	33	GD	60 min 2 times/week	2.5	swim	ESE, SPA
Jeon et al. (2005)	Korea	RCT	253	68.5 ± 0.15	Health	253	RG	60 min 3 times/week	12	None	GDS, Balance
Eyigor et al. (2009)	Türkiye	RCT	37	72.35 ± 10.35	Health	37	TFD	60 min 2 times/week	2	None	SF-36, GDS, BBS
Ayari et al. (2023)	France	RCT	23	78.00 ± 7.00	MCI	16	AD	60 min /week	4	aerobics	MMSE, GDS-15, NPI-R
Adam et al. (2016)	Malaysia	RCT	84	70.87 ± 8.19	MCI	42	PPD	60 min 2 times/week	1.5	Relaxation exercise	HADS, QOL-AD, MMSE
Dos Santos et al. (2023)	Brazil	RCT	35	72.1 ± 1.9	Health	35	DG	50 min 2 times/week	3	None	POMS
Wang et al. (2020)	China	RCT	66	60–70	MCI	47	CSD	40 min 3 times/week	3	Maintain daily activities	MoCA, MMSE, GDS, BBS, PCS, MCS
Zhang et al. (2023)	China	RCT	76	72.30 ± 5.92	Health	39	CSD	60 min 5 times/week	4	Maintain daily activities	Depression, Emotion, Sleep, MMSE, SPPB
Vankova et al. (2014)	Prague	RCT	162	68.55 ± 4.35	MCI	149	BD	60 min/week	3	None	GDS-15
Hola et al. (2024)	Czech Republic	RCT	77	69.00 ± 0.32	Health	64	CD	60 min 2 times/week	3	Maintain daily activities	GDS, Cognitive T-scores

Dance Intervention Types: CSD = Chinese square dance; AD = Aerobic dance; GD = Group dance; RG = Rhythmic gymnastics; TFD = Turkish folk dance; PPD = Poco Poco dance; DG = Dance games; BD = Ballroom dance; CD = Creative dance; CT = Control Group. Assessment Scales: SECF = SECF Cognitive Scale; HAMA = Hamilton Anxiety Scale; HAMD = Hamilton Depression Scale; GDS-30 = Geriatric Depression Screening Scale (assesses depressive symptoms in elderly subjects); MoCA/MoCA-P = Montreal Cognitive Assessment Scale/Concordance Version (MoCA-P) (scores > 26); SF-36 = Psychological Assessment Scale; ESE = Korean version of the Exercise Self-Efficacy Scale; SPA = Korean version of the Social Physiological Anxiety Scale; BBS = Berg Balance Scale (used to evaluate balance ability in the elderly); MMSE = Mini-Mental State Examination Scale (scores > 24); NPI-R = Neuropsychiatric Inventory-Revised Scale; HADS = Hospital Anxiety and Depression Scale; QOL-AD = Quality of Life in Alzheimer’s Disease Scale; SPPB = Short Physical Performance Battery (assesses physical performance). Other Terms: MCI = Mild cognitive impairment; Balance = Balance ability; Depression = Depressive symptoms; Emotion = Emotional well-being.

### 3.3 Bias risk assessment results

Two researchers independently assessed the risk of bias in the included studies using the Cochrane Handbook for Systematic Reviews of Interventions’ Risk of Bias Assessment Tool for Randomised Controlled Trials (RoB 2.0). The assessment process followed the RoB 2.0 guidance, covering five main domains comprising six items: risk of bias in randomisation, risk of bias due to deviation from the allocated intervention, risk of bias due to missing outcome data, risk of bias in outcome measurement, risk of bias due to selective reporting of outcomes, and other sources of bias. Each item was assigned a distinct score based on the assessed risk of bias: low risk of bias was denoted by “+”; high risk of bias by “−“; and insufficient information to determine risk of bias by “!”.

**Figure 2:**
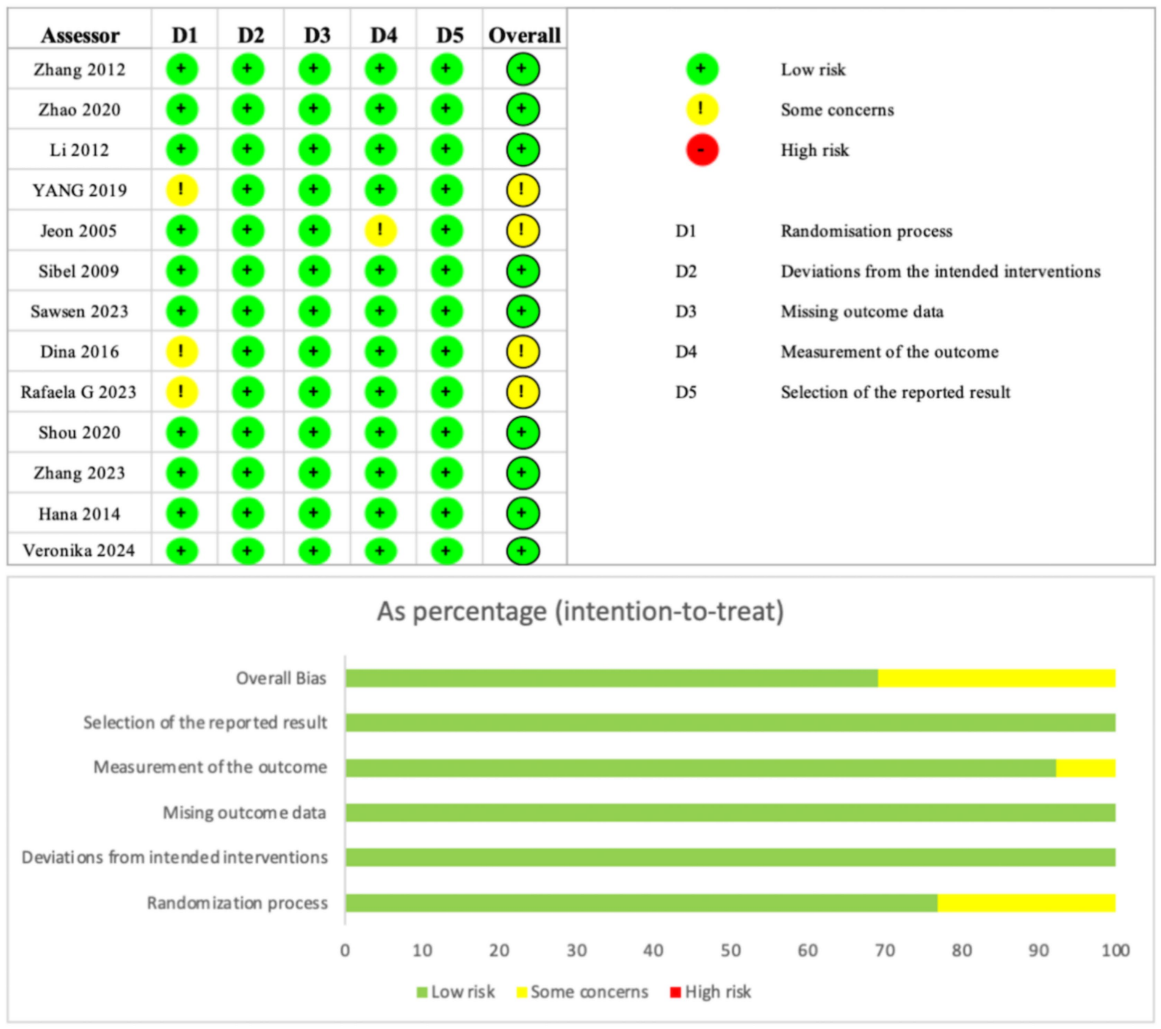
Risk of bias assessment for the included studies using the Cochrane risk-of-bias tool for randomized trials.

Thirteen articles were included in this study. Of these, nine studies were judged to be at low risk of bias, four at moderate risk (some concerns), and none at high risk. The most common sources of moderate risk were deviations from the intended interventions (D2) and selection of the reported result (D5), as shown in the percentage bar chart (Figure 2). Detailed risk of bias assessment results are presented in Figure 2.

These ratings indicate an overall low to moderate risk of bias across the included studies. The presence of moderate risk in some studies, particularly in domains related to intervention adherence and outcome reporting, may reduce the certainty of the evidence (e.g., downgrading from high to moderate certainty per GRADE criteria). This could lead to potential overestimation or underestimation of treatment effects, especially in network meta-analysis where indirect comparisons rely on study quality. To mitigate this, sensitivity analyses excluding moderate-risk studies are recommended to assess the robustness of the findings.

### 3.4 Primary outcome measure

#### 3.4.1 Depression

**Figure 3:**
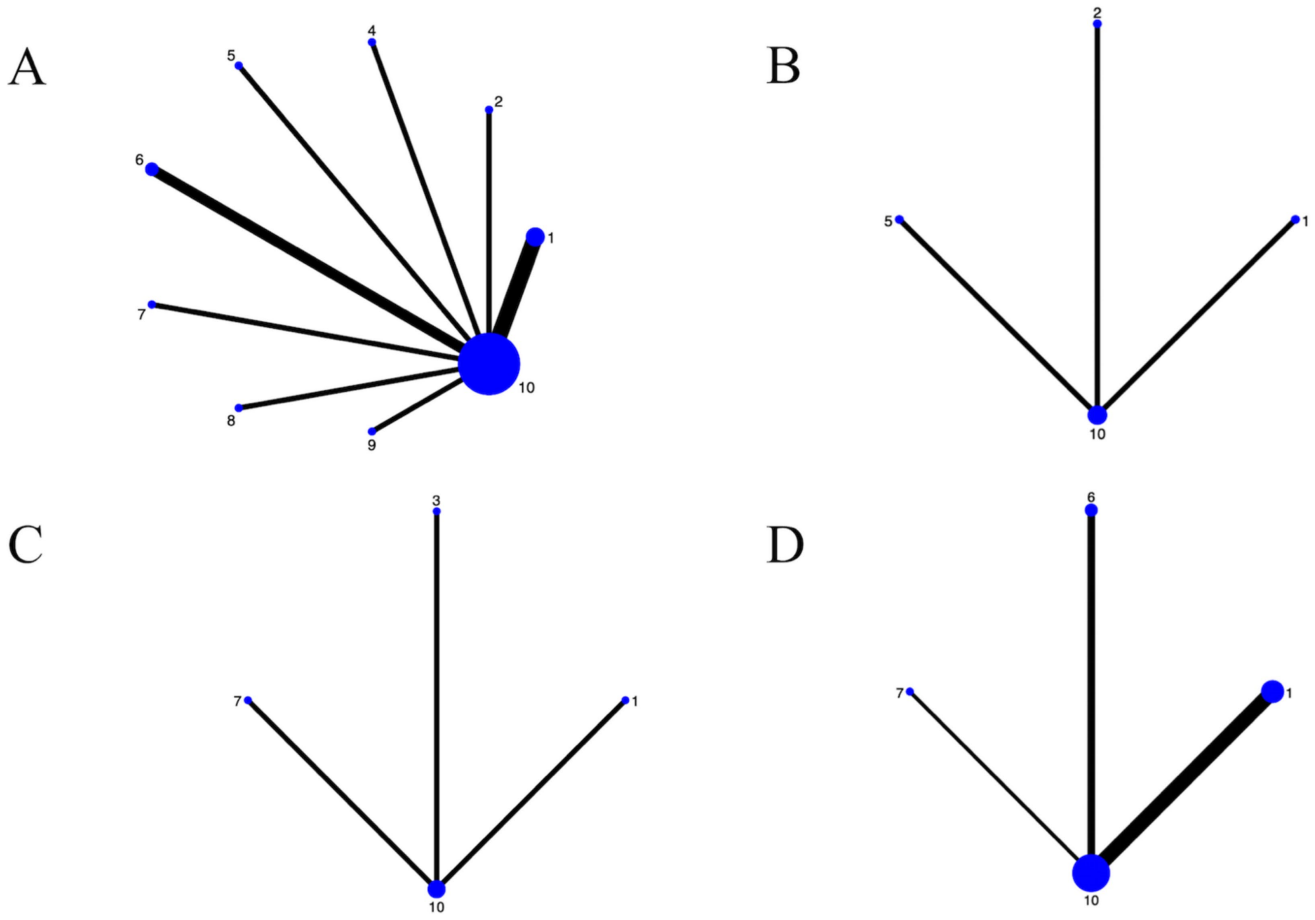
For the network diagrams included in the study. **(A)** Depression; **(B)** Well-being; **(C)** Anxiety; **(D)** Cognitive function; 1: Chinese square dance; 2: Aerobic dance; 3: Group dance; 4: Rhythmic gymnastics; 5: Turkish folk dance; 6: Creative dance; 7: Poco Poco dance; 8: Dance games; 9: Ballroom dance; 10: Control group.

The NMA evaluated the comparative effects of nine dance interventions on depression in older adults, incorporating data from multiple randomized controlled trials. The network plot (Figure 3A) illustrated the structure of the evidence network, comprising nine dance interventions (Chinese square dance, aerobic dance, rhythmic gymnastics, Turkish folk dance, creative dance, Poco dance, dance games, ballroom dance, and group dance) connected through direct and indirect comparisons, with control group as the reference. The network demonstrated moderate connectivity, with thicker lines indicating more direct head-to-head trials, and no isolated nodes, ensuring a cohesive analysis.

**Figure 4:**
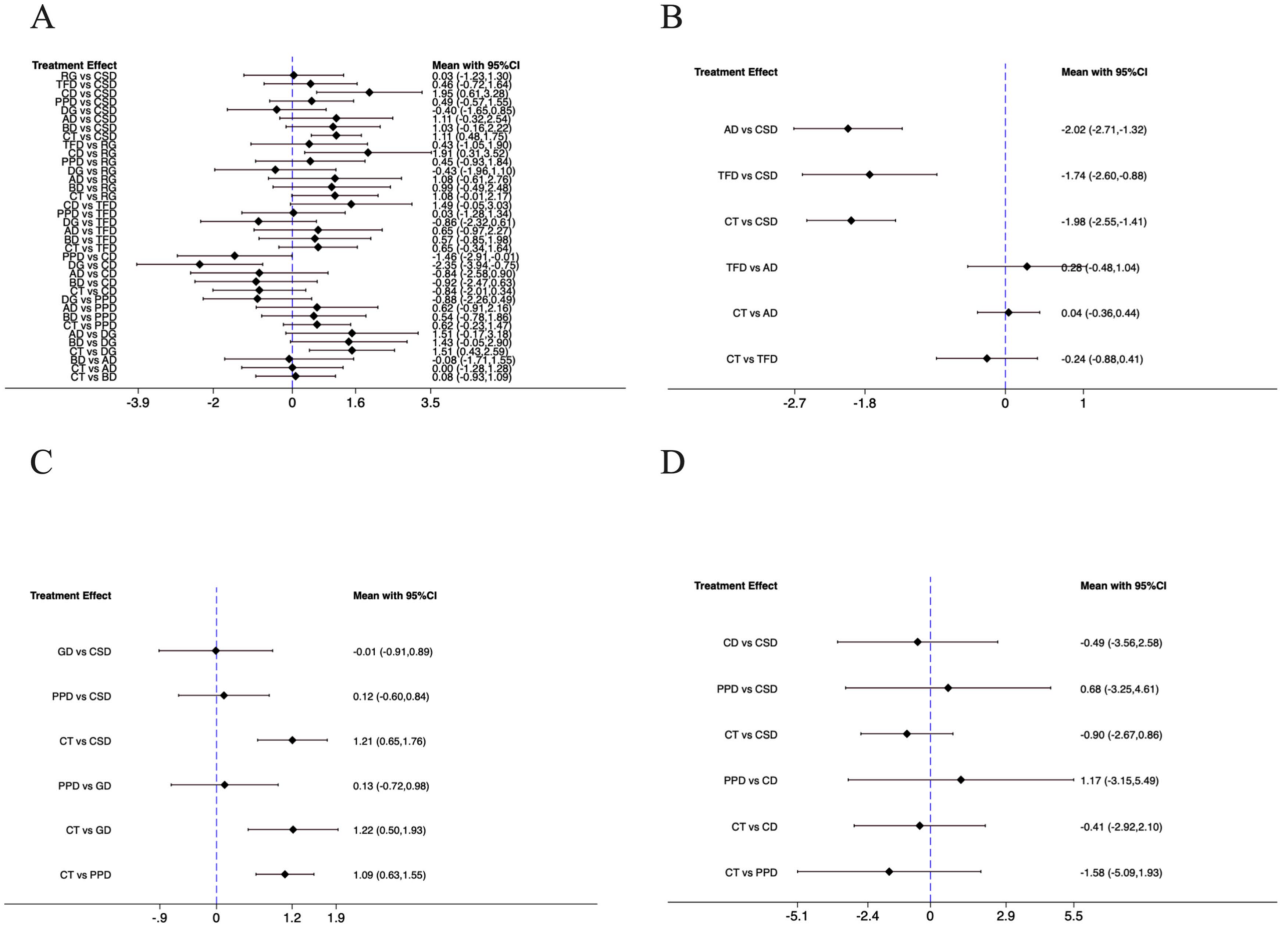
For the forest plots included in the study. **(A)** Depression; **(B)** Well-being; **(C)** Anxiety; **(D)** Cognitive function; CSD, Chinese square dance; AD, Aerobic dance; GD, Group dance; RG, Rhythmic gymnastics; TFD, Turkish folk dance; CD, Creative dance; PPD, Poco Poco dance; DG, Dance games; BD, Ballroom dance; CT, Control group.

The forest plot (Figure 4A) illustrated pairwise comparisons of dance interventions for depressive symptoms using SMD with 95% confidence intervals (CI). The SMD was calculated based on Hedges’ g method. In any comparison of Intervention A versus Intervention B, the SMD is defined as (mean of A—mean of B)/pooled standard deviation. Since lower scores indicate better outcomes, a negative SMD signifies that A is more effective than B, while a positive SMD indicates that B is more effective than A. Consequently, if the effect size and its 95% CI fall entirely to the left of the null line (SMD = 0), it suggests that A is superior; if they fall entirely to the right, it indicates that B is superior. For instance, Poco dance vs. Creative dance showed SMD = −1.46 (95% CI, −2.91, −0.01), indicating that Poco dance was significantly more effective rather than creative dance in reducing depression (*p* < 0.05). Similarly, Dance games vs. Creative dance yielded SMD = −2.35 (95% CI, −3.94, −0.75), confirming the superiority of dance games (*p* < 0.05). In contrast, Chinese square dance demonstrated outstanding efficacy, significantly outperforming creative dance (SMD = 1.95, 95% CI: 0.61, 3.28) in alleviating depressive symptoms and surpassing control group (SMD = 1.11, 95% CI: 0.48, 1.75). Similarly, dance games demonstrated robust efficacy, significantly outperforming conventional treatment (SMD = 1.51, 95% CI: 0.43, 2.59). Conversely, creative dance yielded significantly poorer outcomes than both distinct dance interventions: Chinese square dance and rhythmic gymnastics (SMD = 1.91, 95% CI: 0.31, 3.52).

**Figure 5:**
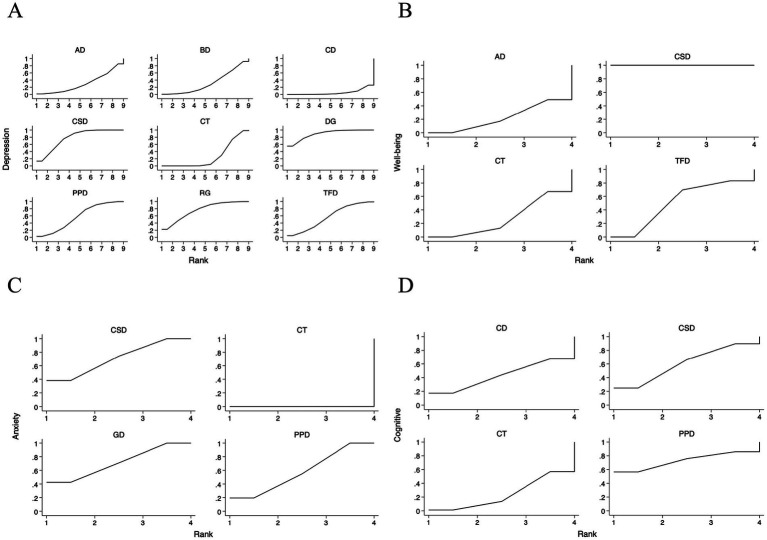
For the sucra sorting chart included in the study. **(A)** Depression; **(B)** Well-being; **(C)** Anxiety; **(D)** Cognitive function; CSD, Chinese square dance; AD, Aerobic dance; GD, Group dance; RG, Rhythmic gymnastics; TFD, Turkish folk dance; CD, Creative dance; PPD, Poco Poco dance; DG, Dance games; BD, Ballroom dance; CT, Control group.

The SUCRA ranking plot (Figure 5A) and associated cumulative probability curves provided an exploratory hierarchy of the relative effectiveness of the interventions. SUCRA values of different dance interventions to depression, well-being, anxiety and cognitive function in Table 2. Based on these metrics, dance games had the highest probability of being the most effective treatment (SUCRA = 89.3%; PrBest = 55.6%), followed by Chinese square dance (SUCRA = 78.5%) and rhythmic gymnastics (SUCRA = 74.6%). In contrast, creative dance (SUCRA = 5.5%) and the control group (SUCRA = 25.8%) showed the lowest probabilities of being optimal treatments. The resulting relative ranking was dance games > Chinese square dance > rhythmic gymnastics > Turkish folk dance > Poco Poco dance > ballroom dance > aerobic dance > control group> creative dance. It is critical to interpret this order as a probabilistic estimate rather than a definitive hierarchy, as the network meta-analysis did not establish statistically significant differences between most of the top-ranked interventions.

**Table 2:** SUCRA values of different dance interventions to depression, well-being, anxiety, and cognitive function.

Outcome measure	CSD	AD	RG	TFD	CD	PPD	DG	BD	GD	CT
Depression	0.785	0.303	0.746	0.570	0.055	0.566	0.893	0.323	/	0.258
Well-being	1.000	0.506	/	0.392	/	/	/	/	/	0.101
Anxiety	0.711	/	/	/	/	0.578	/	/	0.711	0.00
Cognitive	0.603	/	/	/	0.430	0.728	/	/	/	0.240

CSD, Chinese square dance; AD, Aerobic dance; GD, Group dance; RG, Rhythmic gymnastics; TFD, Turkish folk dance; PPD, Poco Poco dance; DG, Dance games; BD, Ballroom dance; CD, Creative dance; CT, Control group.

**Figure 6:**
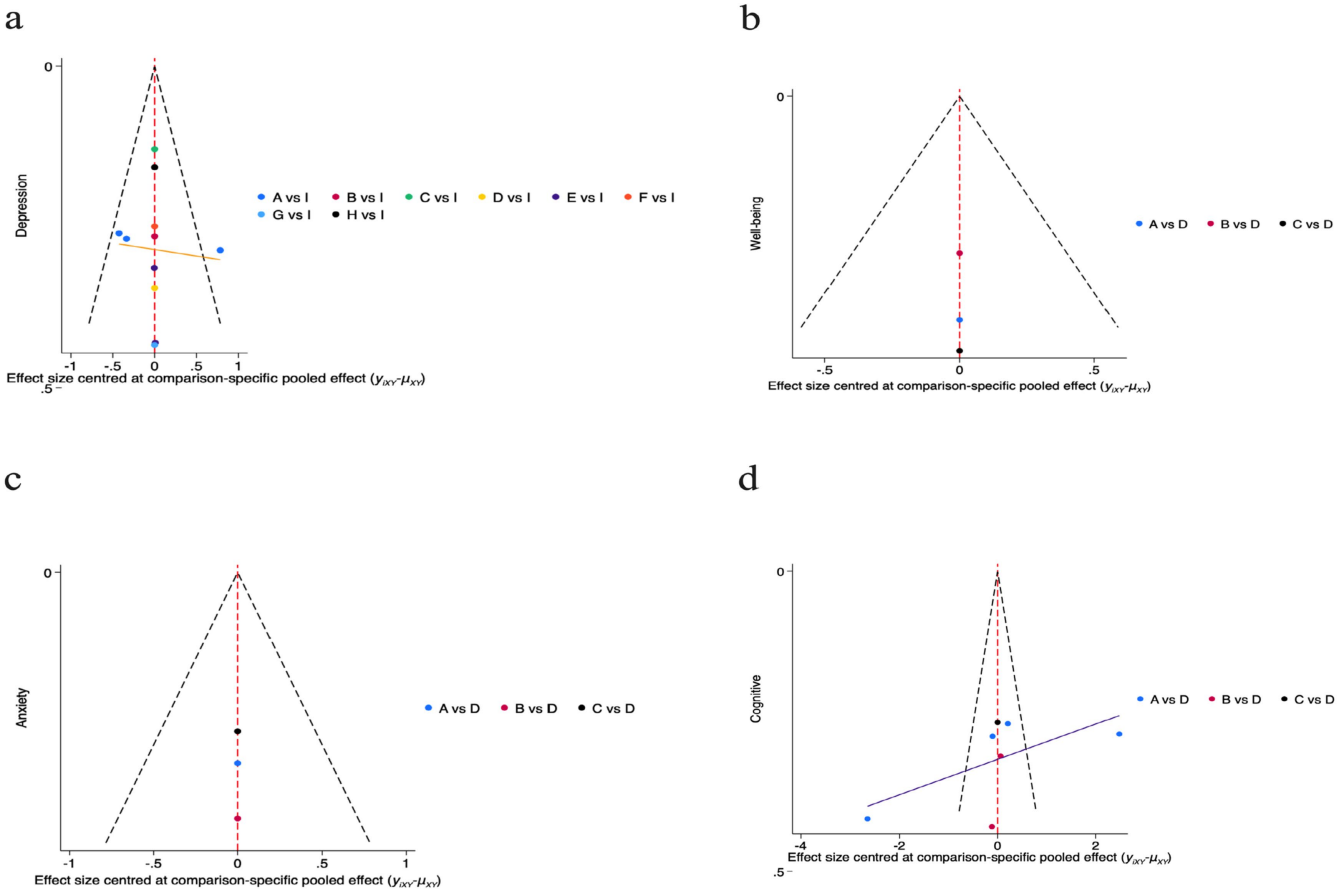
For the funnel plots included in the study. **a**: Depression; A**(a)**: Chinese square dance; B**(a)**: Aerobic dance; C**(a)**: Rhythmic gymnastics; D**(a)**: Turkish folk dance; E**(a)**: Creative dance; F**(a)**: Poco Poco dance; G**(a)**: Dance games; H**(a)**: Ballroom dance; I**(a)**: Control group; **b**: Well-being; A**(b)**: Chinese square dance; B**(b)**: Aerobic dance; C**(b)**: Turkish folk dance; D**(b)**: Control group; **c**: Anxiety; A**(c)**: Chinese square dance; B**(c)**: Group dance; C**(c)**: Poco Poco dance; D**(c)**: Control group; **d**: Cognitive function; A**(d)**: Chinese square dance; B**(d)**: Creative dance; C**(d)**: Poco Poco dance; D**(d)**: Control group.

To assess the potential for small-study effects and publication bias on depressive outcomes, we generated funnel plots centered at the comparison-specific pooled effect (Figure 6A). Visual inspection indicated an approximately symmetrical distribution of effect sizes around the null line, with all estimates residing within the pseudo 95% CI. This pattern suggests a low likelihood of substantial publication bias, meaning that the pooled effect estimates from our analysis are less likely to be artificially inflated due to the systematic non-publication of small, non-significant studies. Consequently, the clinical implications derived from these findings—such as the estimated benefits of one intervention over another—can be viewed with a greater degree of confidence regarding their validity. Nevertheless, these results must be interpreted with caution due to the considerable clinical heterogeneity inherent in the included interventions and the limited number of studies available for specific comparisons. The symmetry observed does not definitively rule out the possibility of bias.

#### 3.4.2 Well-being

The NMA examined the relative effects of dance interventions on well-being outcomes in older adults, drawing from relevant randomized controlled trials. The network plot (Figure 3B) depicted the evidence network with 3 interventions (Aerobic dance, Turkish folk dance and Chinese square dance) and control group, linked via direct and indirect evidence, using no exercise as the comparator. The plot showed limited connectivity with nodes labeled 1, 2, 5, and 10, forming a simple star-like structure centered on node 10, indicating sparse direct comparisons and reliance on indirect evidence.

The forest plot (Figure 4B) presents the pairwise comparisons for well-being outcomes using SMD with 95% CI. For this analysis, higher scores indicate better well-being. In any comparison of Intervention A versus Intervention B, the SMD is defined as (mean of A - mean of B). Consequently, a positive SMD value indicates that Intervention A is more effective than B, while a negative SMD indicates that Intervention B is more effective than A. The results demonstrated a consistent pattern where Intervention Chinese square dance was significantly superior to all other interventions. Specifically: aerobic dance vs. Chinese square dance: SMD = −2.02 (95% CI: −2.71, −1.32), indicating Chinese square dance was significantly more effective than aerobic dance. Turkish folk dance vs. Chinese square dance: SMD = −1.74 (95% CI: −2.60, −0.88), indicating Chinese square dance was significantly more effective than Turkish folk dance. Control group vs. Chinese square dance: SMD = −1.98 (95% CI, −2.55, −1.41), indicating Chinese square dance was significantly more effective than control group.

The SUCRA ranking plot (Figure 5B) provided an exploratory hierarchy of interventions for well-being. Chinese square dance was identified as the highest-ranked intervention, with a SUCRA value of 100.0%, a 100.0% probability of being the best treatment, and a mean rank of 1.0. aerobic dance ranked second (SUCRA = 50.6%; Mean Rank = 2.5), followed by Turkish folk dance (SUCRA = 39.2%; Mean Rank = 2.8). The control group had the lowest ranking (SUCRA = 10.1%; Mean Rank = 3.7) (Table 2). The resulting order was: Chinese square dance > aerobic dance > Turkish folk dance > control group. This probabilistic ranking should be viewed with caution, as the limited number of studies and substantial heterogeneity preclude firm conclusions about statistical superiority.

For the well-being outcome, we plotted a funnel plot centered on the pooled effect size with comparison specificity to preliminarily assess small-sample effects (Figure 6B). Visual inspection revealed that the limited number of data points were broadly symmetrically distributed on either side of the zero line, with all falling within the pseudo 95% CI. No clear evidence of publication bias was discernible. However, given the extremely limited number of direct comparisons available within the network for bias assessment (e.g., encompassing only three comparisons), this finding warrants highly cautious interpretation. The current evidence is insufficient to draw definitive conclusions regarding publication bias.

#### 3.4.3 Anxiety

The NMA assessed comparative efficacy of dance interventions on anxiety levels in older adults, utilizing data from included trials. The network plot (Figure 3C) outlined the network geometry with 3 interventions (Chinese square dance, group dance, and Poco Poco dance) and control group, interconnected by direct/indirect paths, referenced against no exercise. Connectivity was limited, with nodes labeled 1, 3, 7, and 10 forming a basic tree structure centered on node 10, indicating few direct comparisons and heavy reliance on indirect evidence.

The forest plot (Figure 4C) presents the pairwise comparisons for anxiety outcomes using SMD with 95% CI. For this analysis, lower scores indicate better outcomes, consistent with the interpretation of depression scores. The results demonstrated a consistent pattern where the control group was significantly less effective than all dance interventions. Specifically: control group vs. Chinese square group: SMD = 1.21 (95% CI: 0.65, 1.76); control group vs. group dance: SMD = 1.22 (95% CI: 0.50, 1.93); control group vs. Poco Poco dance: SMD = 1.09 (95% CI: 0.63, 1.55). For comparisons among the dance interventions, no statistically significant differences were observed. The comparisons of group dance vs. Chinese square dance: SMD = −0.01 (95% CI: −0.91, 0.89), Poco Poco dance vs. Chinese square dance: SMD = 0.12 (95% CI: −0.60, 0.84), Poco Poco dance vs. group dance: SMD = 0.13 (95% CI: −0.72, 0.98) all yielded CI that crossed the null line.

The SUCRA ranking plot (Figure 5C) provided an exploratory hierarchy of interventions for anxiety outcomes. Chinese square dance and group dance were jointly ranked as the most effective interventions, with identical SUCRA values of 71.1% and mean ranks of 1.9. Group dance had a slightly higher probability of being the best treatment (PrBest = 42.8%) compared to Chinese square dance (PrBest = 38.7%). Poco Poco dance ranked third (SUCRA = 57.8%; PrBest = 18.5%; Mean Rank = 2.3), while control group was ranked as the least effective option (SUCRA = 0.0%; PrBest = 0.0%; Mean Rank = 4.0) (Table 2). The resulting exploratory order was: Chinese square dance = group dance > Poco Poco dance > control group. This suggests that Chinese square dance and group dance are likely among the most beneficial options, though the analysis cannot confirm a statistically significant difference between them.

For the anxiety outcome, we plotted a funnel plot centered on the comparison-specific pooled effect size to preliminarily assess small-sample effects (Figure 6C). Visual inspection revealed that the limited number of data points were broadly symmetrically distributed on either side of the zero line, with all falling within the pseudo 95% confidence interval, showing no apparent signs of publication bias.

### 3.5 Secondary outcome measure

#### 3.5.1 Cognitive function

The NMA investigated dance interventions’ effects on cognitive function in older adults, based on trial data. The network plot (Figure 3D) visualized the network, featuring 3 interventions (Chinese square dance, Creative dance and Poco Poco dance) and control group, integrated through comparisons, with no exercise as baseline. The network exhibited limited linkages, with nodes labeled 1, 3, 6, 7, and 10 forming a tree-like structure centered on node 10, indicating sparse direct comparisons and dependence on indirect paths.

The forest plot (Figure 4D) presents the pairwise comparisons for cognitive function outcomes using SMD with 95% CI. For this analysis, higher scores indicate better cognitive function, consistent with the interpretation of well-being scores. While none of the comparisons reached statistical significance, as all CI crossed the null line, the point estimates reveal a consistent and noteworthy trend favoring dance interventions over conventional treatment. This non-significant but consistent trend can be observed in several key comparisons Chinese square dance showed a trend towards superiority over control group (SMD = −0.90 [95% CI: −2.67, 0.86]), creative dance also trended favorably against control group (SMD = −0.41 [95% CI: −2.92, 2.10]), Poco Poco dance demonstrated the strongest trend versus control group (SMD = −1.58 [95% CI: −5.09, 1.93]).

The SUCRA ranking plot (Figure 5D) provided an exploratory hierarchy of the interventions for cognitive function outcomes. Based on the cumulative probabilities, Poco Poco dance had the highest likelihood of being the most effective intervention (SUCRA = 72.8%; Probability of being the best = 56.6%; Mean Rank = 1.8), followed by Chinese square dance (SUCRA = 60.3%; Probability of being the best = 24.8%; Mean Rank = 2.2). Creative dance and the control group ranked third and fourth, respectively (CD: SUCRA = 43.0%, Mean Rank = 2.7; CT: SUCRA = 24.0%, Mean Rank = 3.3) (Table 2). The resulting exploratory ranking was: Poco Poco dance > Chinese square dance > Creative dance > control group. Importantly, the CI for all pairwise comparisons in the cognitive function network were wide and crossed the null line, indicating that this ranking is highly uncertain and should be considered hypothesis-generating.

For cognitive function outcomes, we plotted a funnel plot centered on the comparative specificity pooled effect size to preliminarily assess potential small-sample effects (Figure 6D). Visual inspection revealed data points distributed symmetrically around the zero line, with all falling within the pseudo 95% CI, indicating no apparent signs of publication bias. This finding suggests that the pooled effect size for cognitive function in this network meta-analysis is unlikely to be substantially influenced by small-sample studies. However, it must be emphasized that given the limited number of direct comparisons available for assessment, caution is warranted in interpreting this result. The current graphical evidence does not support the presence of publication bias, but it cannot entirely rule out this possibility.

## 4 Discussion

### 4.1 The impact of dance intervention on depression in older adults

The results of this NMA reveal that dance interventions exert a modest alleviating effect on depressive symptoms in older adults, with no statistically significant differences among the interventions. Dance games and Chinese square dance ranked highest in SUCRA (89.3 and 78.5%, respectively; Table 2), highlighting the potential of interactive, gamified, and rhythmic forms. This aligns with existing literature, where a systematic review and meta-analysis demonstrated that dance interventions significantly reduce depression in older adults with a nearly moderate effect size (Wu et al., 2021). Similarly, another meta-analysis confirmed a statistically significant reduction in depressive symptoms (*p* < 0.01) among older adults engaging in dance, particularly in forms emphasizing social engagement (Kontos et al., 2021). These findings underscore the potential of dance as a multifaceted tool for mental health improvement in aging populations.

Mechanistically, the benefits of dance on depression likely stem from its integration of physical activity, social interaction, and rhythmic stimulation (Lakes et al., 2016), which collectively activate neurochemical pathways involving dopamine and serotonin release, thereby mitigating depressive states (Jie et al., 2025). For dance games, the gamified elements (e.g., virtual rewards and progression) boost motivation and cognitive function engagement, countering apathy in depression (Rüth and Kaspar, 2020). Similarly, the communal aspect of Chinese square dance fosters belonging and emotional regulation, countering isolation—a major depression risk in elders (Li et al., 2025a).

Clinically, these results advocate for the incorporation of accessible dance forms like dance games and Chinese square dance into community programs as cost-effective, non-pharmacological options to improve adherence and mental health. Future studies should focus on high-quality RCTs with direct comparisons, long-term follow-ups, and subgroup analyses by depression severity to optimize strategies.

### 4.2 The impact of dance intervention on well-being in older adults

This NMA highlights the promising role of dance interventions in enhancing well-being among older adults, with Chinese square dance showing the strongest effect (SUCRA 100.0%; Table 2). Although the network has limited connectivity and relies on indirect evidence, pairwise comparisons indicate Chinese square dance significantly outperforms aerobic dance, Turkish folk dance, and controls (SMDs ranging from −1.74 to −2.02). These trends favor culturally resonant and interactive dances. Well-being, as a critical component of overall health in older adults, is influenced by factors such as social engagement, physical activity, and emotional regulation (Charles, 2010). This finding aligns with systematic reviews indicating that dance-based interventions improve multidimensional well-being, encompassing physical, emotional, and cognitive function domains, while reducing symptoms of anxiety and depression and fostering positive life perceptions in the elderly (Christensen et al., 2021). Such corroborative evidence positions dance as a holistic promoter of subjective well-being, particularly in aging populations where social isolation and diminished physical capabilities often erode quality of life.

Mechanistically, the superior performance of Chinese square dance can be attributed to its integration of moderate physical exertion with rich social interactions, which likely stimulates endorphin release, enhances self-efficacy, and strengthens interpersonal bonds—key pathways for elevating well-being (Li and Liang, 2024; Xiang and Jiang, 2025). Studies have shown that dance interventions improve physical function, balance, and overall quality of life by promoting mobility and emotional uplift, with group dynamics amplifying these effects through shared experiences and cultural resonance (Haynes et al., 2023). The cultural accessibility of Chinese square dance, rooted in community participation, further explains its mechanism in boosting positive affect and countering age-related psychological decline (Heyang et al., 2024).

Clinically, these insights advocate for the integration of accessible, interactive dances like Chinese square dance into elderly care protocols, serving as engaging, non-pharmacological strategies to combat social isolation in community settings. By leveraging low-cost, enjoyable activities, such interventions could improve adherence and long-term well-being outcomes. Future research should expand RCTs to include diverse dance modalities, incorporating advanced neuroimaging techniques to elucidate underlying brain mechanisms and dose–response relationships, thereby optimizing tailored interventions for enhanced well-being in older adults.

### 4.3 The impact of dance intervention on anxiety in older adults

This NMA suggests dance interventions mildly reduce anxiety in older adults, with Chinese square dance and group dance tied for top SUCRA (71.1%; Table 2), though no significant differences exist among dances. This is supported by a meta-analysis showing dance’s significant impact on reducing anxiety alongside cognition and depression (Fong Yan et al., 2024). Such consistency positions dance as a viable anxiety management approach in geriatrics.

Mechanistically, dance alleviates anxiety through parasympathetic activation, rhythmic entrainment, and social cohesion, which collectively downregulate stress hormones like cortisol (Bégel et al., 2022). Evidence from meta-analyses reveals that dance movement therapy (DMT) decreases anxiety by enhancing quality of life and emotional expression (Christensen et al., 2021). For Chinese square dance and group dance, communal elements build support networks, countering loneliness—a key anxiety trigger (Xiao and Hilton, 2019).

Clinically, these interventions, especially socially oriented ones, could be embedded in anxiety prevention programs for seniors, leveraging their low-risk profile for broad implementation. Future investigations should utilize multi-arm RCTs to directly compare dance variants, incorporating biomarkers and real-time anxiety metrics to deepen understanding of therapeutic pathways.

### 4.4 The impact of dance intervention on cognitive function in older adults

This NMA indicates dance interventions modestly improve cognitive function in older adults, with Poco Poco dance ranking highest in SUCRA (72.8%) followed by Chinese square dance (60.3%; Table 2), though no significant differences were found. This echoes a meta-analysis where dance improved global cognition and executive function in older adults (Hewston et al., 2021). Additionally, dance therapy has been shown to enhance memory, balance, and overall cognitive function health in aging populations (Balazova et al., 2021). These alignments affirm dance’s role in cognitive preservation.

Mechanistically, dance fosters neuroplasticity by engaging multisensory coordination, music processing, and social interaction, stimulating hippocampal and prefrontal regions (Noguera et al., 2020; Blasi et al., 2025). Meta-analytic evidence indicates significant gains in global cognition, memory, and executive function from dance therapy (Fong Yan et al., 2024). For Poco and Chinese square dance, rhythmic and communal elements enhance sequencing, attention, and self-efficacy.

Clinically, promoting dances like Poco and Chinese square dance could prevent cognitive function decline in lifestyle programs. Future longitudinal RCTs with neuroimaging should validate mechanisms and optimal dosing.

### 4.5 Advantages and prospects of dance intervention in older adults

Dance, as a comprehensive intervention form that integrates physical activity with emotional expression, exhibits significant multidimensional advantages, making it particularly suitable for older adults (Pitluk Barash et al., 2023). Its dual nature as both exercise and art not only promotes aerobic metabolism and enhances physical fitness but also mobilizes positive emotions through musical rhythm and social interaction, thereby strengthening psychological resilience and social connectedness (Blasi et al., 2025). Empirical evidence from systematic reviews and meta-analyses supports these benefits; for instance, a meta-analysis demonstrated that dance interventions significantly improve physical function, balance, and quality of life in older adults, with effects on muscular strength, endurance, and postural control (Liu et al., 2021). Another review highlighted dance’s role in reducing depression and anxiety symptoms while fostering emotional well-being, attributing this to its holistic engagement of body and mind (Li and Liang, 2024). Clinically, non-pharmacological interventions like dance hold significant value as safe, cost-effective complements to pharmacological treatments for older adults’ mental health. Unlike medications, which may cause side effects (e.g., drowsiness or dependency), dance promotes adherence through enjoyment and social engagement, reducing depression and anxiety, while improving cognition and well-being. In the context of aging populations, dance stands out as a low-cost, sustainable, and non-pharmacological approach that can be widely promoted in non-medical settings, boasting high group acceptability and strong potential for community dissemination due to its enjoyable and inclusive nature.

Looking ahead, the prospects for dance interventions in older adults are promising, with opportunities to further enhance their practicality and accessibility in clinical and public health practices. As global aging accelerates, integrating dance into preventive healthcare could address multifaceted challenges, such as cognitive function decline and social isolation, supported by emerging evidence from online dance programs that show positive impacts on physical health and cognition even in remote settings. Future advancements may involve tailoring dance protocols to individual needs, leveraging technology like virtual reality for broader reach, and conducting large-scale RCTs to establish dose–response relationships and long-term efficacy. By bridging cultural and generational gaps, dance interventions hold the potential to become a cornerstone of geriatric mental health strategies, promoting active aging and overall well-being on a societal scale.

### 4.6 Limitations

Despite providing preliminary evidence of the positive impact of dance interventions on the mental health of older adults, this study is subject to several limitations that warrant careful consideration. Firstly, the limited number of included studies, with some dance types represented by only one or a few trials, introduces uncertainty into the pooled effects and may compromise the robustness of the network meta-analysis findings. This scarcity is a common challenge in dance intervention research, as highlighted in a systematic review and meta-analysis on dance for psychological and cognitive function health in older adults, which noted that small sample sizes and limited trial numbers often lead to wide CI and potential overestimation of effects.

Secondly, substantial heterogeneity across the included studies poses a significant challenge. The studies vary considerably in design (e.g., randomized controlled trials versus quasi-experimental designs), types of dance interventions (e.g., Chinese square dance, ballroom, or creative dance), measurement instruments (e.g., different scales for assessing depression or anxiety), intervention frequency and duration, and participant demographics. This methodological variability complicates the analytical models, potentially introduces bias into the aggregated results, and limits the generalizability of the findings to broader older adult populations. For instance, differences in dance styles and assessment tools may lead to inconsistent effect sizes, making it difficult to draw definitive conclusions applicable across diverse cultural or clinical contexts. Notably, 5 out of 13 included studies (approximately 38.5%) originated from China, potentially introducing a geographic bias. This emphasis on Chinese square dance may limit generalizability to other cultures, where social and communal aspects of dance vary. Future research should include diverse populations and culturally adapted interventions to enhance broader applicability.

Additionally, the absence of long-term follow-up in most included studies limits insights into the sustained effects and underlying mechanisms of dance interventions. Many trials focused solely on short-term outcomes, failing to capture enduring psychological benefits or potential relapse over time. This gap is echoed in existing reviews on dance for older adults’ well-being, where short-term trials predominate and overlook long-term adherence and maintenance of mental health improvements. As a result, the external validity of our results is constrained, particularly for informing long-term public health strategies.

The implications of these findings extend to future research and clinical practice, offering pathways to refine and expand dance interventions. To address the identified limitations, upcoming studies should prioritize standardized designs, such as multi-arm randomized controlled trials with larger sample sizes and consistent outcome measures, to better explore differential impacts of various dance types on specific psychological indicators like depression, anxiety, well-being, and cognitive function. Integrating biological markers, including functional brain imaging (e.g., fMRI) to reveal neural mechanisms, could provide deeper insights.

## 5 Conclusion

This study systematically evaluated the effects of dance interventions on depression, well-being, anxiety, and cognitive function in older adults through NMA. The results indicate that dance interventions overall exert a positive influence on improving psychological health in this population, particularly in alleviating depressive symptoms and enhancing well-being, where trends were more pronounced. Chinese square dance and creative dance demonstrated higher potential intervention benefits across multiple outcome indicators, with promising application prospects due to their accessibility and social engagement features. The consistent positive trends in intervention groups underscore dance as a safe, feasible, and readily promotable non-pharmacological tool that contributes to promoting psychological and cognitive function health in older adults.

From a public health standpoint, dance interventions align with the proactive prevention principles of healthy aging strategies, with their group-based implementation fostering social support networks that mitigate isolation and enhance community cohesion. The evidence from this NMA provides empirical grounds for incorporating dance therapy into community mental health service systems, offering substantial practical guidance for diverse intervention approaches to achieve healthy aging. To build on these insights, future research should prioritize large-scale, rigorously designed randomized controlled trials to validate the findings, exploring the specific influences of different dance types, intervention dosages, and underlying mechanisms on efficacy. Additionally, adopting mixed methods designs that integrate quantitative evaluations with qualitative interviews, while focusing on long-term follow-up data beyond 6 months, will be crucial to assess the sustainability of intervention effects. Ultimately, this study advances the evidence base for dance as a versatile, inclusive strategy in geriatric care, paving the way for optimized mental health promotion in older populations worldwide.
